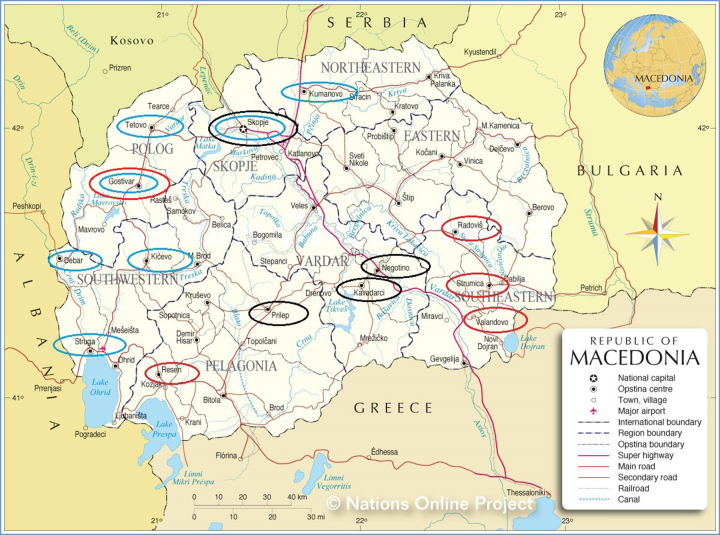# Mitochondrial DNA control region analysis of three ethnic groups in the Republic of Macedonia

**DOI:** 10.1016/j.fsigen.2014.06.013

**Published:** 2014-11

**Authors:** Renata Jankova-Ajanovska, Bettina Zimmermann, Gabriela Huber, Alexander W. Röck, Martin Bodner, Zlatko Jakovski, Biljana Janeska, Aleksej Duma, Walther Parson

**Affiliations:** a Institute of Forensic Medicine, Criminalistic and Medical Deontology, Medical Faculty, University “Ss. Cyril and Methodius”, Skopje, Macedonia; b Institute of Legal Medicine, Innsbruck Medical University, Innsbruck, Austria; c Penn State Eberly College of Science, University Park, PA, USA

**Keywords:** mtDNA, EMPOP, Haplogroups, Albanians, Turks, Romanies

## Abstract

A total of 444 individuals representing three ethnic groups (Albanians, Turks and Romanies) in the Republic of Macedonia were sequenced in the mitochondrial control region. The mtDNA haplogroup composition differed between the three groups. Our results showed relatively high frequencies of haplogroup H12 in Albanians (8.8%) and less in Turks (3.3%), while haplogroups M5a1 and H7a1a were dominant in Romanies (13.7% and 10.3%, respectively) but rare in the former two. This highlights the importance of regional sampling for forensic mtDNA databasing purposes. These population data will be available on EMPOP under accession numbers EMP00644 (Albanians), EMP00645 (Romanies) and EMP00646 (Turks).

## 1 Introduction

According to demographic data from the register in 2002 the population of the Republic of Macedonia is 2,022,547 with 64.2% ethnical Macedonian, 25.2% ethnical Albanians, 3.9% ethnical Turks, 2.7% ethnical Romanies and a small percentage of other ethnic groups. People from different ethnic communities rarely have marriages between each other due to their national and religious determination. Whether or not this has an effect on the distribution of mitochondrial lineages has yet not been studied for Macedonia. An earlier study described mitochondrial (mt)DNA control region variation for ethnical Macedonians, which brought a similar haplogroup distribution to other West-Eurasian populations [1]. Here, we describe mtDNA control region variation in carefully selected samples of the three other major ethnic groups (148 Albanians, 150 Turks and 146 Romanies) and thus add a total of 444 high quality mtDNA lineages to the body of world-wide mtDNA database. The data will also be made available for forensic searches via EMPOP [2] under accession numbers EMP00644 (Albanians), EMP00645 (Romanies), and EMP00646 (Turks).

## 2 Materials and methods

### 2.1 Samples and DNA extraction

This study was reviewed and approved by the ethics commission of the University “St.Cyril and Methodius” (study classification number 03-5904/2 from 01.02.13, session number XXVI). All participants (*N* = 444) gave their written consent before a buccal swab was taken. Study participants were sampled from different geographic locations in the Republic of Macedonia (Fig. S1), Albanians derived mostly from the western part, Turks originated from the eastern, western and southern parts and Romanies derived from the central and northern parts of the country. DNA was extracted using the QIAamp mini kit (Qiagen, Hilden, Germany) following the manufacturer's recommendations.

### 2.2 Amplification, sequencing and data analysis

PCR amplification and mtDNA control region sequencing were carried out following the EMPOP protocol [3] updated in [4]. Nucleotide sequences were analysed and interpreted using Sequencher (Version 5.1, Gene Codes Corporation) and aligned relative to the rCRS [5] following phylogenetic alignment rules defined in [6]. The mtDNA haplotypes were determined using EMMA [7] based on Phylotree (www.phylotree.org; Build 16; [8]). The random match probability was calculated as sum of squares of the haplotype frequencies [9]. Genetic diversity indices were calculated using the ARLEQUIN software (Version 3.5) [10]. C-Stretch length variants in HVS-I (around 16,193), HVS-II (around 309) and HVS-III (around 573) were ignored for calculating random match probabilities and genetic diversity indices.

## 3 Results and discussion

The mtDNA control region sequence analysis in three Macedonian ethnic groups consisting of 444 individuals (148 Albanians, 150 Turks and 146 Romanies) showed 108 different haplotypes (73%) in Albanians, 100 (66.7%) in Turks and 64 (43.8%) in Romanies, respectively (Tables 1 and S1). Thereof, 87 (80.6%), 74 (74%) and 42 (65.6%) were unique and haplotype diversity was 0.983, 0.986 and 0.966 respectively (Table 1). AMOVA was performed taking into consideration the following published datasets: Macedonia [1], Greece [11], Cyprus [11], Hungarian Ashkenazi [12], Hungarian Baranya Romany [13], Hungarians from Budapest [13], Romanian Csango [14] and Romanian Szekely [14]. Fst comparison, pairwise differences and shared haplotypes are given in ESM 1.

**Table 1:** Diversity measures for control region haplotypes (16024-576) from three Macedonian ethnic groups.

	Albanians	Turks	Romanies
*N*	148	150	146
Number of haplotypes	108	100	64
Number of unique haplotypes	87	74	42
Haplotype diversity	0.990	0.992	0.972
s.e. of haplotype diversity	0.0035	0.0019	0.0048
Mean number of pairwise differences	7.264 ± 3.421	6.641 ± 3.152	8.221 ± 3.833

The distribution of observed lineages differed between the three investigated populations (Table 2). Albanians showed a relatively high abundance of hg H12 lineages (8.8%) that were generally rare elsewhere, 1.3% in northern Greeks [11] and 3% in Orthodox Macedonians [1]. Romanies showed high frequencies of hgs H7a1a (10.3%) and M5a1 (13.7%) that is common in the South Asian phylogeny [15]. This emphasizes the requirement of regional databases when assessing haplotype frequencies in a forensic context.

**Table 2:** Distribution of selected haplogroups in three Macedonian ethnic groups.

Haplogroup	Albanians (*N* = 148)		Turks (*N* = 150)		Romanies (*N* = 146)	
	*n*	%	*n*	%	*n*	%
H7a1a	1	0.67	1	0.66	15	10.27
H12	13	8.78	5	3.33	0	0
M5a1*	1	0.67	0	0	20	13.7
M5a1b	0	0	0	0	18	12.3
W	9	6.08	7	4.66	0	0
X2e1	0	0	0	0	11	7.5

## Figures and Tables

**Figure:**